# *Lactiplantibacillus plantarum* HOKKAIDO strain drives alveolar macrophage-dependent antiviral protection against respiratory infection

**DOI:** 10.3389/fimmu.2026.1929200

**Published:** 2026-09-21

**Authors:** Mari Ikehata, Tomohiro Okagawa, Hayato Nakamura, Maho Inoue, Masami Morimatsu, Kumiko Yoshimatsu, Takamitsu Tsukahara, Kentaro Abe, Mitsuru Honma, Naoya Maekawa, Yasuhiko Suzuki, Shiro Murata, Kazuhiko Ohashi, Satoru Konnai

**Affiliations:** 1 Department of Disease Control, Faculty of Veterinary Medicine, Hokkaido University, Sapporo, Japan; 2 Business Development Unit, FASMAC Co. Ltd., Atsugi, Japan; 3 One Health Research Center, Hokkaido University, Sapporo, Japan; 4 Department of Applied Veterinary Science, Faculty of Veterinary Medicine, Hokkaido University, Sapporo, Japan; 5 Institute for Genetic Medicine, Hokkaido University, Sapporo, Japan; 6 Kyoto Institute of Nutrition & Pathology, Inc., Uji, Japan; 7 Hokkaido Research Station, Snow Brand Seed Co., Ltd., Naganuma, Japan; 8 Institute for Vaccine Research and Development (HU-IVReD), Hokkaido University, Sapporo, Japan; 9 Veterinary Research Unit, International Institute of Zoonosis Control, Hokkaido University, Sapporo, Japan

**Keywords:** alveolar macrophages, antiviral effects, gut microbiota, *Lactiplantibacillus plantarum* HOKKAIDO strain, respiratory infection, systemic immune responses

## Abstract

Calves’ immature immune systems make them susceptible to various infections. In particular, diarrhea and respiratory diseases impair growth and increase mortality, resulting in substantial economic losses in the cattle industry. Probiotics are living microorganisms that confer health benefits to the host and are increasingly recognized as promising preventive and supportive therapies for calf infections. Our previous study revealed that *Lactiplantibacillus plantarum* HOKKAIDO strain (Lp-HKD) reduces intestinal pathology and diarrhea severity in bovine rotavirus-infected calves. We also demonstrated that Lp-HKD activates bovine immune cells, including monocytes and T cells, and exerts an antiviral effect by inducing antiviral cytokines *in vitro*. Based on these findings, we hypothesized that Lp-HKD might also protect against respiratory viral infections. In this study, we investigated the antiviral effects of Lp-HKD against respiratory infection in a mouse model. DBA/2 mice were intragastrically administered Lp-HKD or phosphate-buffered saline for 7 days prior to intranasal inoculation with Sendai virus (SeV). Lp-HKD administration improved survival and reduced weight loss after infection. At 7 days post-infection, SeV levels in the lungs were significantly lower in Lp-HKD–treated mice. Notably, SeV infection significantly decreased alveolar macrophage numbers in control mice, but not in Lp-HKD–treated mice. Furthermore, Lp-HKD treatment reduced the production of inflammatory cytokines and chemokines in bronchoalveolar lavage fluid. Lp-HKD administration was also associated with modest changes in the gut microenvironment, including altered abundance of specific bacterial taxa and increased microbial richness. Conversely, the depletion of alveolar macrophages abolished the protective effect of Lp-HKD, demonstrating that these cells are essential mediators of Lp-HKD antiviral activity. Collectively, these findings indicate that Lp-HKD modulates host immune responses and reduces the severity of viral respiratory infection in mice, underscoring its potential as a viable preventive strategy against viral respiratory diseases.

## 1 Introduction

Calf infections adversely affect growth and can even result in mortality, leading to severe economic losses to the cattle industry (1–3). In particular, diarrhea and respiratory diseases are the most significant causes of death from infectious diseases in calves (4), requiring effective prevention and control measures. Probiotics are live microorganisms such as lactic acid bacteria that confer beneficial effects on the host when administered in adequate amounts (5, 6). *Lactiplantibacillus plantarum* HOKKAIDO strain (Lp-HKD) is a plant-derived probiotic lactic acid bacterium that was originally isolated from vegetables traditionally fermented in wells in Hokkaido, Japan (7). Consistent with other plant-derived lactic acid bacteria, Lp-HKD does not establish long-term intestinal colonization and instead exerts its effects through transient interaction with the host. Although Lp-HKD may enhance antiviral immune responses in humans (8, 9), we previously demonstrated that feeding milk replacer supplemented with high Lp-HKD concentrations alleviated diarrhea and intestinal tissue damage caused by bovine rotavirus infection (10). Furthermore, culture assays of bovine immune cells revealed that Lp-HKD activates monocyte and T cell functions and exerts antiviral activity by inducing antiviral cytokines such as interferon (IFN)-γ (11). Therefore, we hypothesized that Lp-HKD may not only ameliorate viral diarrhea but also a broader range of viral infectious diseases.

The gut–lung axis has attracted increasing attention as a bidirectional, complex communication network between the intestinal tract and the respiratory system, emerging as a novel target for preventive and therapeutic strategies against respiratory diseases (12). Accumulating evidence indicates that certain probiotic strains ameliorate viral respiratory infections by modulating host immunity (13). Accordingly, the use of probiotics may be a promising adjunctive strategy in the management of respiratory infections. However, despite these advances, the effects of Lp-HKD as a probiotic with both immunostimulatory and antiviral properties in the context of viral respiratory infections remain unexplored.

Therefore, in this study, we investigated the protective effects of intragastric administration of Lp-HKD against viral respiratory infection and elucidated the underlying immune mechanisms, particularly investigating pulmonary immune cells and the gut microenvironment. DBA/2 mice, known to be highly susceptible to respiratory viral pathogens (14), were intragastrically administered Lp-HKD for 7 days prior to intranasal inoculation with Sendai virus (SeV; murine parainfluenza virus type 1) that causes acute respiratory disease (15). Subsequently, we assessed systemic immune responses, disease outcomes after viral infection, pulmonary immune responses, and changes in the gut microenvironment.

## 2 Materials and methods

### 2.1 Preparation of Lp-HKD and SeV

Lp-HKD (Food Processing Research Center, Hokkaido Research Organization, Ebetsu, Japan; Japanese patent No. 3925502) was cultured in MRS broth (BD Biosciences, San Jose, CA, USA) at 37°C for 24 h and collected by centrifugation at 5,800 × g for 15 min at 10°C. The bacteria were washed twice with PBS and finally resuspended in PBS. The bacteria were plated on MRS agar plate (BD Biosciences) and anaerobically incubated at 37°C for 17–24 h and colony-forming unit (CFU) were counted. The live Lp-HKD was stored at 4°C and used for further experiments within 7 days. SeV (MN strain) was grown in embryonated chicken eggs, stored at -80°C and thawed prior to infection as previously described (16). Virus levels were expressed as tissue culture infectious dose (TCID_50_).

### 2.2 Administration of Lp-HKD and SeV infection in mice

Eight-week-old female DBA/2 mice (Japan SLC, Shizuoka, Japan and Japan CLEA, Tokyo, Japan) were used in this study because of their high susceptibility to SeV infection (14). All experimental procedures were carried out with the approval of the Ethics Committee of the Faculty of Veterinary Medicine, Hokkaido University (approval number: 23-0039; approval date: March 28, 2023). All procedures in this study adhered to the ARRIVE guidelines (17). A one-week acclimation period was provided after introduction. The sample size was determined based on preliminary experiments and previously published studies using similar models, which employed comparable group sizes. Animals were allocated to experimental groups based on availability rather than formal randomization. Mice were intragastrically administered 100 µl of Lp-HKD (1 × 10^9^ CFU) or PBS (control) daily from 7 days prior to SeV inoculation. Regarding SeV inoculation, mice were anesthetized using a triple-combination anesthetic consisting of medetomidine (Nippon Zenyaku Kogyo Co., Ltd., Fukushima, Japan), midazolam (Nichi-Iko Pharmaceutical Co., Ltd., Toyama, Japan and Sandoz Group AG, Basel, Switzerland), and butorphanol (Meiji Animal Health Co., Ltd., Kumamoto, Japan), administered intraperitoneally, and then inoculated intranasally with 25 µl of SeV (1.0 × 10^3^ TCID_50_). After SeV inoculation, atipamezole (Nippon Zenyaku Kogyo Co., Ltd.) was administered intraperitoneally as an antagonist. Mice were observed for up to 14 days following SeV inoculation, during which administration of Lp-HKD continued throughout the entire infection period. Survival and body weight were recorded daily for 14 days after SeV inoculation. Mice were sacrificed at some time points during the experiment, and the spleen, bronchoalveolar lavage fluid (BALF), lung, small intestine, and cecal feces were collected. Investigaters were not blinded during sample collection due to logistical constraints. To minimize bias, data analysis was performed using predefined criteria. Details of the processing procedures for each sample are described below.

### 2.3 Processing of the spleen

After sampling, the spleen weight was measured first. Subsequently, single-cell suspensions were prepared from the spleen using the Minute Single Cell Isolation Kit (Invent Biotechnologies, Inc., Plymouth, MN, USA) according to the manufacturers’ protocols. Half of the single-cell suspension was used for flow cytometric analysis, and the other half was used for quantitative reverse transcription polymerase chain reaction (qRT-PCR) assay.

### 2.4 Collection of the BALF

The BALF was collected from the trachea using 24-gauge indwelling catheter (Terumo Corporation, Tokyo, Japan). A small incision was made in the trachea to permit insertion of 24-gauge lavage tube. The lungs were rinsed with 500 µl of physiological saline (Otsuka Pharmaceutical Co., Ltd., Tokyo, Japan) for 3 times, and the BALF was collected in 1.5 ml low-protein-binding tube. The BALF was then centrifuged at 18,700 × g for 5 min at 4°C. The supernatant was stored at -30°C for quantification of cytokines and chemokines.

### 2.5 Processing of the lung

Single-cell suspensions were prepared from the lung using the Minute Single Cell Isolation Kit (Invent Biotechnologies, Inc.) according to the manufacturer’s protocols, and used for flow cytometric analysis.

### 2.6 Counting of the Peyer’s patches

After collecting the small intestine, the number of Peyer’s patches was counted macroscopically.

### 2.7 Flow cytometric analysis of immune cells

The prepared spleen and lung cells were harvested and blocked with anti-CD16/CD32 monoclonal antibody (mAb) (BD Biosciences) at 25°C for 15 min. After washing, the cells were stained at 4°C for 20 min with different antibodies; FITC-conjugated anti-CD45 mAb (BioLegend, San Diego, CA, USA), PE-conjugated anti-Ly6G mAb (BioLegend), PerCP/Cy5.5-conjugated anti-CD3 mAb (SONY, Tokyo, Japan), PE-Cy7-conjugated anti-CD45R/B220 mAb (BioLegend), APC-conjugated anti-CD335 (NKp46) mAb (BioLegend), PE-conjugated anti-CD11b mAb (BioLegend), PerCP/Cy5.5-conjugated anti-CD115 mAb (BioLegend), PE-Cy7-conjugated anti I-A/I-E mAb (BioLegend), APC-conjugated anti-CD11c mAb (SONY), and Fixable Viability Dye eFluor 780 (Thermo Fisher Scientific). Gating strategy and representative dot plots are shown in **Figures 1** and **2**. The number of cells were counted using CountBright Absolute Counting Beads (Thermo Fisher Scientific) according to the manufacturer’s protocols. Antibody dilution and cell washing were performed with PBS containing 1% bovine serum albumin (Sigma-Aldrich, St. Louis, MO, USA) and 2 mM ethylenediaminetetraacetic acid (Dojindo Molecular Technologies, Kumamoto, Japan). The stained cells were washed and analyzed immediately using the FACSLyric Flow Cytometry System (BD Biosciences).

**Figure 1:**
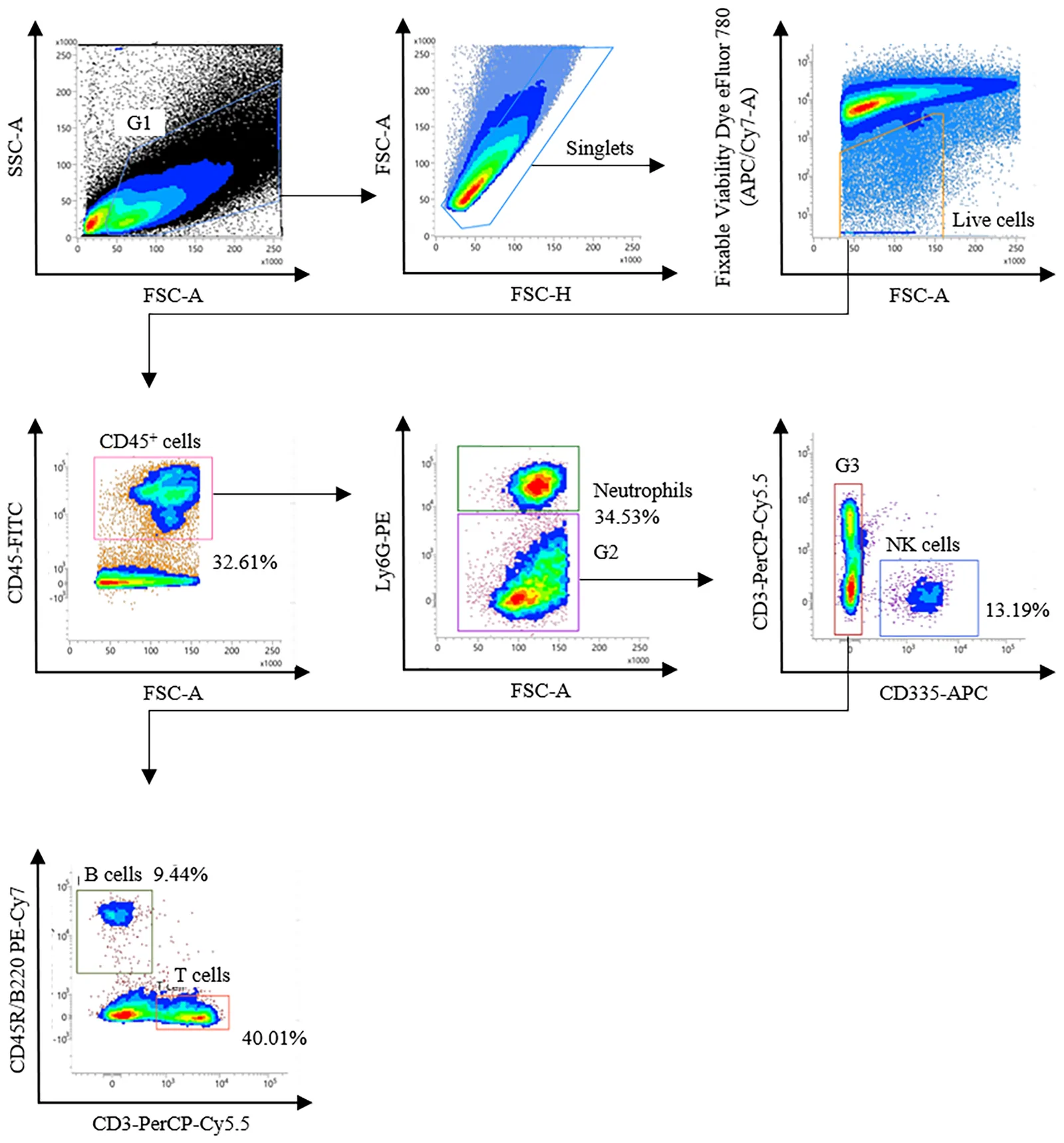
Gating strategy and representative dot plots for analyses of CD45^+^ cell, neutrophils, NK cells, B cells, and T cells. The immune cells were analyzed by flow cytometry in spleen and lung cells. The CD45^+^ cells were sub-classified as Neutrophils (Ly6G+), NK cells (Ly6G-CD335+), B cells (Ly6G-CD335-CD45R/B220+), and T cells (Ly6G-CD335-CD3+).

**Figure 2:**
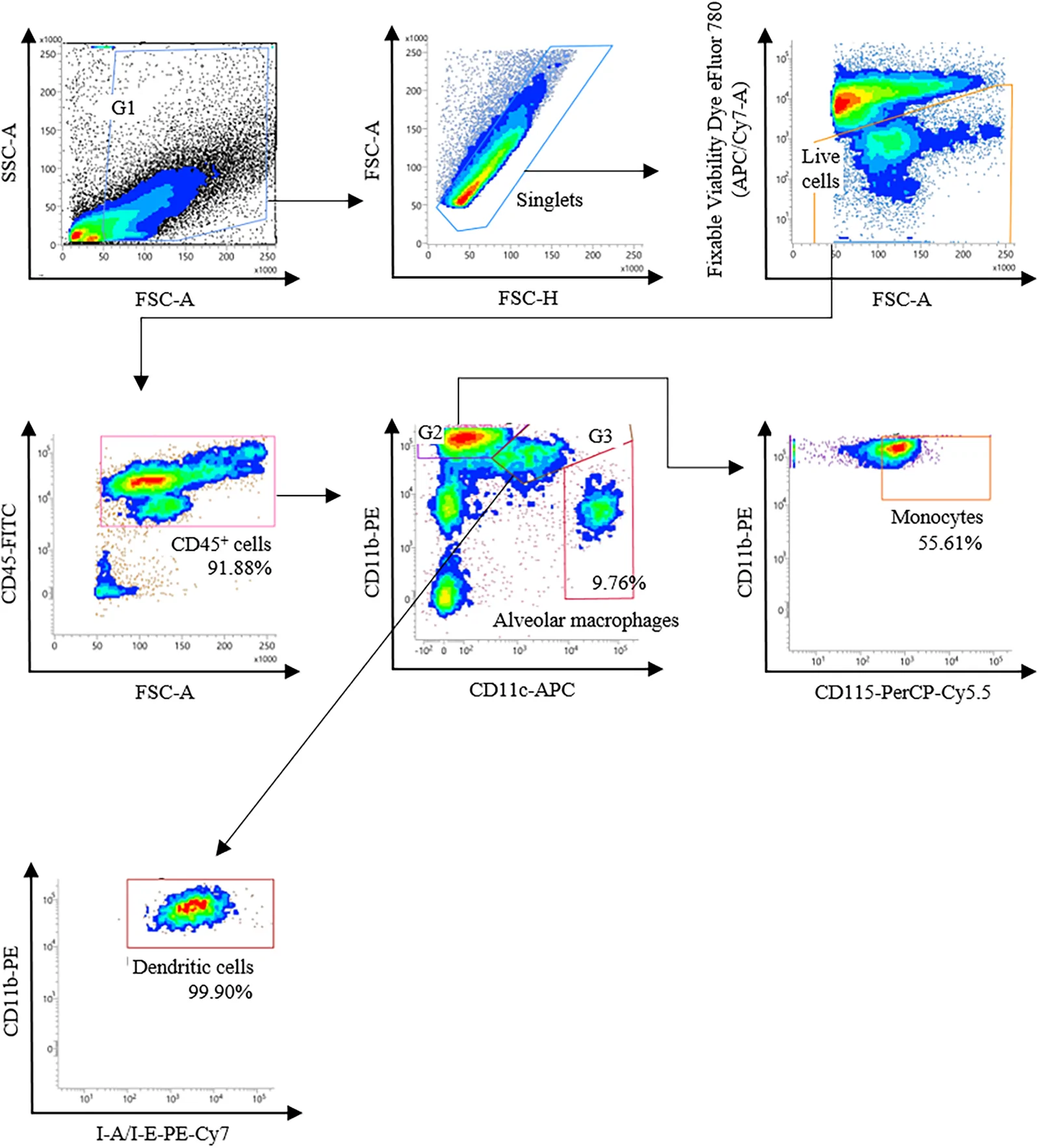
Gating strategy and representative dot plots for analyses of CD45^+^ cells, alveolar macrophages, monocytes, and dendritic cells. The immune cells were analyzed by flow cytometry in spleen and lung cells. The CD45^+^ cells were sub-classified as Alveolar macrophages (CD11b-CD11c) (only in the lung), Monocytes (CD11b+CD11c-CD115+), and Dendritic cells (CD11b+CD11c+I-A/I-E+).

### 2.8 Quantification of cytokines by qRT-PCR

mRNA expression for IFN-γ and tumor necrosis factor (TNF)-α was assessed using qRT-PCR. Total RNA was extracted from the spleen cells using the Direct-zol RNA Miniprep Plus (Zyomo Research, Irvine, CA, USA) and purified using the Monarch RNA Cleanup Kit (New England Biolabs, Ipswich, MA, USA) according to the manufacturers’ instructions. cDNA was synthesized using PrimeScript Reverse Transcriptase (Takara Bio, Shiga, Japan) with an Oligo dT primer, following the manufacturer’s instructions. The resulting cDNA was used as the template for the subsequent qPCR. qPCR was performed using TB Green Premix DimerEraser (Takara Bio) with the QuantStudio 12K Flex Real-Time PCR System (Thermo Fisher Scientific). Housekeeping gene β-actin was used as an internal control. The specific primer sequences were as follows: *Actb* (forward: 5’–AGAGGGAAATCGTGCGTGAC–3’, reverse: 5’–CAATAGTGATGACCTGGCCGT–3’), *IFN-γ* (forward: 5’–GCGTCATTGAATCACACCTG–3’, reverse: 5’–TGAGCTCATTGAATGCTTGG–3’), and *TNF-α* (forward: 5’–CTGAACTTCGGGGTGATCGG–3’, reverse: 5’ –GGCTTGTCACTCGAATTTTGAGA–3’). Amplification was performed in 40 cycles, with initial incubation at 95°C for 30 sec; each cycle consisted of denaturation at 95°C for 3 sec, annealing at 55°C for 30 sec, and extension at 72°C for 30 sec. Cytokine expression levels were determined by relative quantification using the 2^-ΔΔCt^ method.

### 2.9 Lung viral RNA levels

Total RNA extraction, purification, and cDNA synthesis were performed as described above. The resulting cDNA was used as the template for the subsequent qPCR. The gene expression levels of SeV and β-actin were determined from cDNAs with TaqMan Gene Expression Assays (Mr04269880_mr and Mm02619580_g1, respectively) and TaqMan Fast Advanced Master Mix (Thermo Fisher Scientific) through the QuantStudio 12K Flex Real-Time PCR System (Thermo Fisher Scientific). Amplification was performed in 40 cycles, with initial incubation at 50°C for 2 min and at 95°C for 20 sec; each cycle consisted of denaturation at 95°C for 1 sec, annealing and extension at 60°C for 20 sec. Cytokine expression levels were determined by relative quantification using the 2^-ΔΔCt^ method. The available lung samples were prioritized for RNA quantification and immunological analyses, and the remaining material was insufficient for infectious titer assays. Therefore, viral burden was validated using qRT-PCR measurements.

### 2.10 Quantification of cytokines and chemokines by multiplex assay

Concentration of cytokines and chemokines in the BALF were analyzed using Bio-Plex Pro Mouse Cytokine Grp I panel 23-Plex (Bio-Rad, Hercules, CA, USA), and analysis was performed using the MAGPIX system (Luminex Corporation, Austin, TX, USA).

### 2.11 Gut microbiota and organic acid analyses of cecal feces

After collection, cecal feces were stored at -80°C until analyses. For gut microbiota analysis, DNA was extracted from cecal fecal samples according to the method as previously described (18). Using the extracted DNA, library preparation was conducted following “16S Metagenomic Sequencing Library Preparation” protocol (Illumina, Inc., San Diego, CA, USA). Sequencing was performed on a MiSeq platform (Illumina) according to the manufacturer’s instructions. The resulting sequence data were analyzed using Quantitative Insights Into Microbial Ecology 2 (Pycogent). For organic acid analysis, cecal feces were initially subjected to pretreatment and then analyzed by ion-exclusion high-performance liquid chromatography as previously described (19). The concentrations of succinate, lactate, formate, *iso*-butyrate, *n*-butyrate, acetate, propionate, *iso*-valerate, and *n*-valerate were measured.

### 2.12 Depletion of alveolar macrophages from mice lungs

To deplete alveolar macrophages in the lung, mice were intranasally administered 50 µl of clodronate liposomes (Katayama Chemical Industries Co., Ltd., Osaka, Japan) under anesthesia. As a control, empty liposomes were administered. All administrations were performed 4 days prior to SeV infection. Subsequently, all mice inoculated intranasally with 25 µl of SeV (0.6 × 10^3^ TCID_50_) and their survival and body weight were recorded daily, as described above.

### 2.13 Statistical analysis

All data were statistically analyzed using GraphPad Prism 10.1.0 (GraphPad Software, San Diego, CA, USA). Differences were determined using Student’s *t*-test and Welch’s *t*-test for two-group comparisons. For the correlation analysis, Pearson correlation analysis was applied. A *p*-value of less than 0.05 was considered statistically significant.

## 3 Results

### 3.1 Activation of systemic immune responses by Lp-HKD administration

We first investigated the effects of Lp-HKD administration on systemic immune responses via splenic immunological analyses. After 7 days of intragastric administration of Lp-HKD or PBS (control), spleens were collected (**Figure 3A**). The specimens were weighed and normalized to body weight, and the measurements were compared between the two groups. The relative spleen weight was higher in the Lp-HKD group than in the control group (**Figure 3B**). Similarly, flow cytometric analysis revealed significantly higher splenic leukocyte numbers in the Lp-HKD group than in the control group (**Figure 3C**). Furthermore, analysis of cytokine gene expression showed higher IFN-γ and TNF-α expression levels in the Lp-HKD group than in the control group (**Figure 3D**). Altogether, these results suggest that Lp-HKD administration may enhance systemic immune responses.

**Figure 3:**
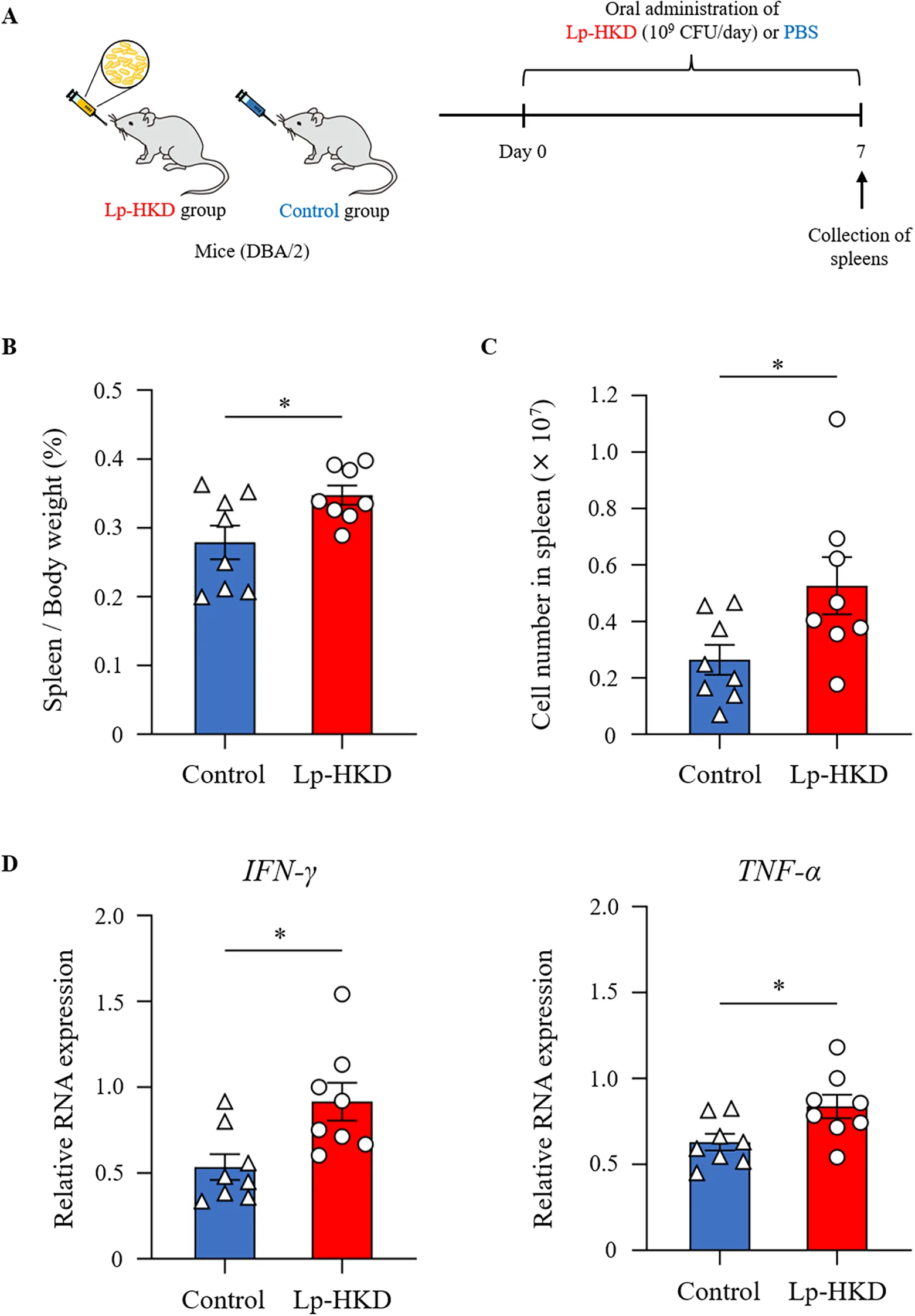
Lp-HKD-induced activation of systemic immune responses in the spleen. **(A)** Schematic overview of the experimental design. DBA/2 mice (*n* = 8/group) were intragastrically administered 100 µl of Lp-HKD (1 × 10^9^ CFU/mouse) or PBS (control) daily for 7 days, after which the mice were sacrificed and the spleen was collected. **(B)** Spleen/body weight ratio (%). **(C)** Absolute number of splenic leukocytes. **(D)** Gene expression levels of IFN-γ and TNF-α in the spleen. Statistical significance was determined by the Welch’s *t*-test. *p < 0.05.

### 3.2 Effects of Lp-HKD administration on the health of SeV-infected mice

Next, we examined the protective effects of Lp-HKD against viral respiratory infection. Mice were intragastrically administered Lp-HKD or PBS (control) daily, starting 7 days prior to viral challenge and continuing throughout the experimental period. On Day 0, all mice were intranasally infected with SeV (10^3^ TCID_50_). Survival and body weight were monitored daily for 14 days following infection (**Figure 4A**). In the control group, the survival rate decreased to 50% at Day 10 post-infection, and all mice had died by Day 11. In contrast, the Lp-HKD group had maintained an 80% survival rate at Day 14 (**Figure 4B**). Regarding body weight changes, both groups showed similar weight loss rates through Day 6. However, from Day 7 onward, mice in the Lp-HKD group began to recover, and the differences in body weight changes between the two groups became significant on Days 9 and 10 (**Figure 4C**). Thus, Lp-HKD administration contributed to prolonged survival and promoted recovery from weight loss following SeV infection.

**Figure 4:**
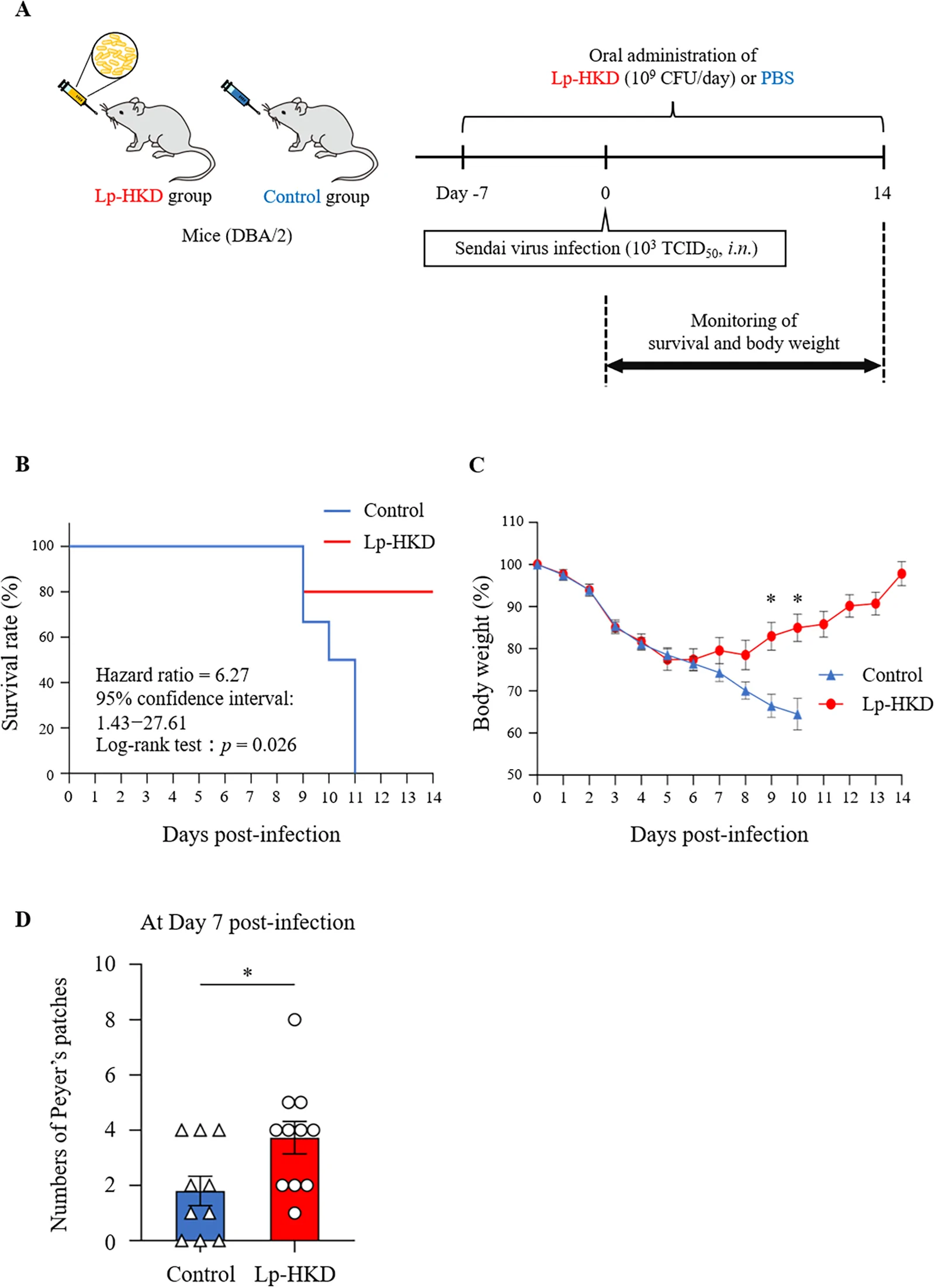
Lp-HKD-induced protective effects against SeV infection. **(A)** Outline of the experimental scheme. DBA/2 mice (Lp-HKD group, *n* = 5; control group, *n* = 6) received daily intragastric administration of 100 µl of Lp-HKD (1 × 10^9^ CFU/mouse) or PBS (control) beginning 7 days before SeV infection (10^3^ TCID_50_) and continuing until Day 14 post-infection. **(B)** Survival rates after SeV infection. **(C)** Body weight changes expressed as percentages relative to body weight on Day 0. **(D)** Number of Peyer’s patches in the small intestine. Statistical significance was determined by the Log-rank test **(A)**, Student’s *t*-test **(B)** and the Welch’s *t*-test **(C)**. * *p* < 0.05.

At Day 7 post-infection, mice were sacrificed and the small intestine was collected for counting Peyer’s patches. The number was significantly higher in the Lp-HKD group than in the control group (**Figure 4D**). Notably, no significant difference was observed between the groups at Day 0 prior to inoculation in the number of Peyer’s patches (**Supplementary Figure S1**). Previous studies have found that nutritional deficiency significantly reduces the number of Peyer’s patches (20). Therefore, Lp-HKD administration may be linked to subtle differences in the host’s physiological condition during SeV infection; however, the biological significance of this observation remains uncertain.

### 3.3 Modulation of pulmonary immune responses by Lp-HKD administration during SeV infection

Because mice in the Lp-HKD group began to show signs of body weight recovery from Day 7 post-infection (**Figure 4C**), subsequent analyses were focused on this time point. Mice were sacrificed on Day 7 post-infection, and the lungs were collected for further analysis (**Figure 5A**). Viral RNA levels in the right lung decreased significantly in the Lp-HKD group compared with the control group (**Figure 5B**). Flow cytometry analysis showed no significant differences in the absolute numbers of dendritic cells, monocytes, neutrophils, B cells, T cells, or NK cells (**Figure 5C**). Interestingly, the number of alveolar macrophages, which was similar between the groups at Day 0 (**Supplementary Figure S2**), was higher in the Lp-HKD group than in the control group. Furthermore, a negative correlation was observed between viral RNA levels and the absolute number of alveolar macrophages (**Figure 5D**). These results collectively indicate that alveolar macrophages play an important role in Lp-HKD–mediated protection against SeV infection.

**Figure 5:**
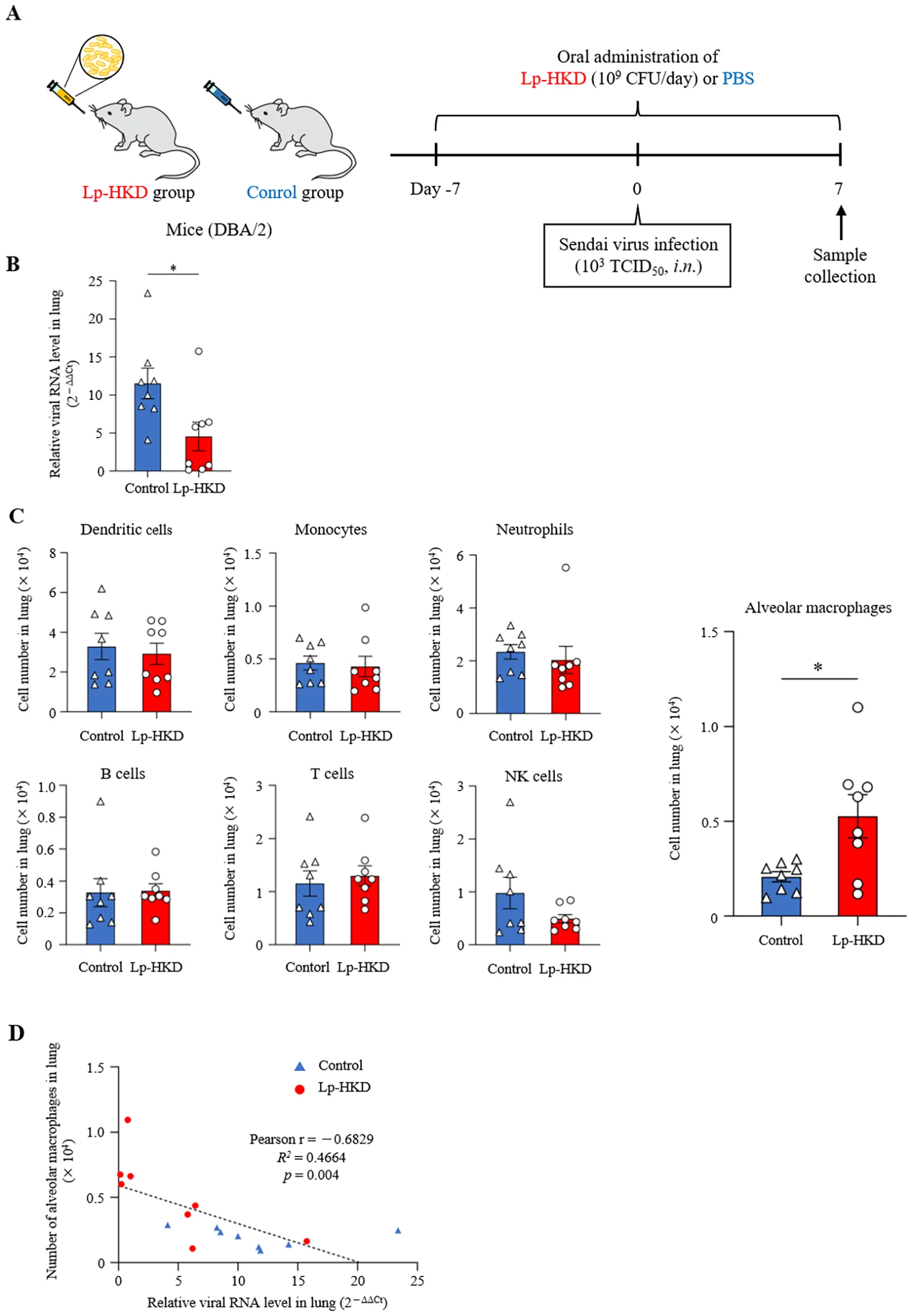
Lp-HKD-mediated modulation of pulmonary immune responses during SeV infection. **(A)** Overview of the experimental scheme. DBA/2 mice (*n* = 8/group) received daily intragastric administration of 100 µl of Lp-HKD (1 × 10^9^ CFU/mouse) or PBS (control) beginning 7 days before SeV infection (10^3^ TCID_50_) and continuing until Day 7 post-infection. Mice were sacrificed on Day 7 post-infection, and the lungs were collected for analysis. **(B)** Viral RNA levels in the right lung. **(C)** Absolute numbers of immune cell populations in the left lung. **(D)** Correlation between viral RNA levels and the absolute number of alveolar macrophages. Statistical significance was determined by the Welch’s *t*-test. The relationship between the variables was evaluated using Pearson correlation analysis. *p < 0.05.

### 3.4 Reduction of BALF inflammatory cytokines and chemokines during SeV infection by Lp-HKD administration

Furthermore, we collected BALF to measure cytokine and chemokine levels at Day 7 post-infection using the Bio-Plex Pro Mouse Cytokine 23-plex assay. The Lp-HKD group tended to show lower levels of inflammatory cytokines and chemokines in BALF than the PBS group (**Figure 6A**). Specifically, among these factors, the levels of four inflammatory cytokines, namely, IFN-γ, TNF-α, interleukin (IL)-12 p40, and IL-17A, were significantly lower in the Lp-HKD group than in the control group (**Figure 6B**). The levels of four other chemokines—KC (CXCL1), MCP-1 (CCL2), MIP-1α (CCL3), and RANTES (CCL5)—also significantly decreased in the Lp-HKD group compared with the control group (**Figure 6C**). Overall, Lp-HKD administration was associated with reduced lung inflammation.

**Figure 6:**
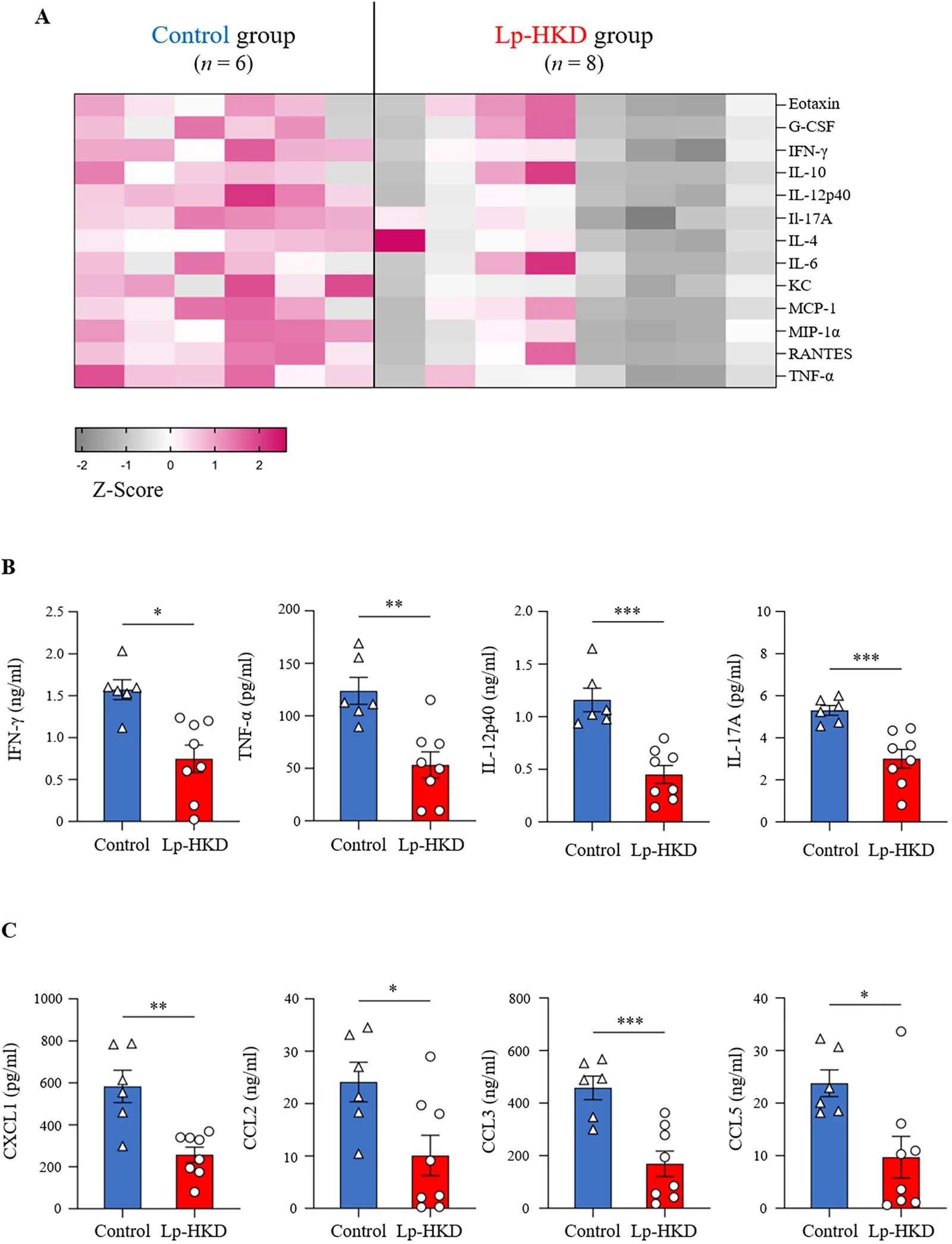
Lp-HKD-mediated reduction of BALF inflammatory cytokines and chemokines during SeV infection. **(A)** Heatmap of inflammatory cytokine and chemokine levels in the BALF (Lp-HKD group, *n* = 8; control group, *n* = 6). **(B)** Concentrations of inflammatory cytokines in the BALF. **(C)** Concentrations of chemokines in the BALF. Statistical significance was determined by the Welch’s *t*-test. *p < 0.05, **p < 0.01, ***p < 0.001.

### 3.5 Impact of Lp-HKD administration on the gut microenvironment

To evaluate the impact of Lp-HKD administration on the gut microenvironment, we next analyzed the gut microbiota and organic acid levels in cecal feces collected at Day 3 post-infection. Because cecal feces were nearly unobtainable at Day 7 post-infection, especially in the control group, where disease progression severely reduced fecal output, the gut microenvironment was analyzed using samples collected at Day 3 post-infection. Regarding gut microbiota composition, the two groups differed significantly in the relative abundance of several bacterial taxa in cecal feces, including members of the *Lactobacillaceae* family, which showed marked variation between the groups (**Supplementary Figure S3A**; **Supplementary Table 1**). Furthermore, we compared the alpha diversity between the Lp-HKD and control groups using the Chao1 and Shannon entropy indices (**Figure 7A**). The Chao1 index indicated greater microbial richness in the Lp-HKD group than in the control group, whereas the Shannon entropy index did not differ significantly between the groups. However, beta diversity analysis using principal component analysis (PCA) revealed no distinct clustering of gut microbiota communities between groups, which was corroborated by PERMANOVA results (*p* = 0.075; **Figure 7B**). Furthermore, regarding organic acid concentrations, *n*-butyrate, acetate, and propionate levels were significantly lower in the Lp-HKD group than in the control group (**Supplementary Figure S3B**), but no significant differences were observed for those of other organic acids (data not shown). Therefore, while Lp-HKD administration did not substantially alter the overall structure of the gut microbiota, it did affect the diversity of certain taxa and modulate microbial richness.

**Figure 7:**
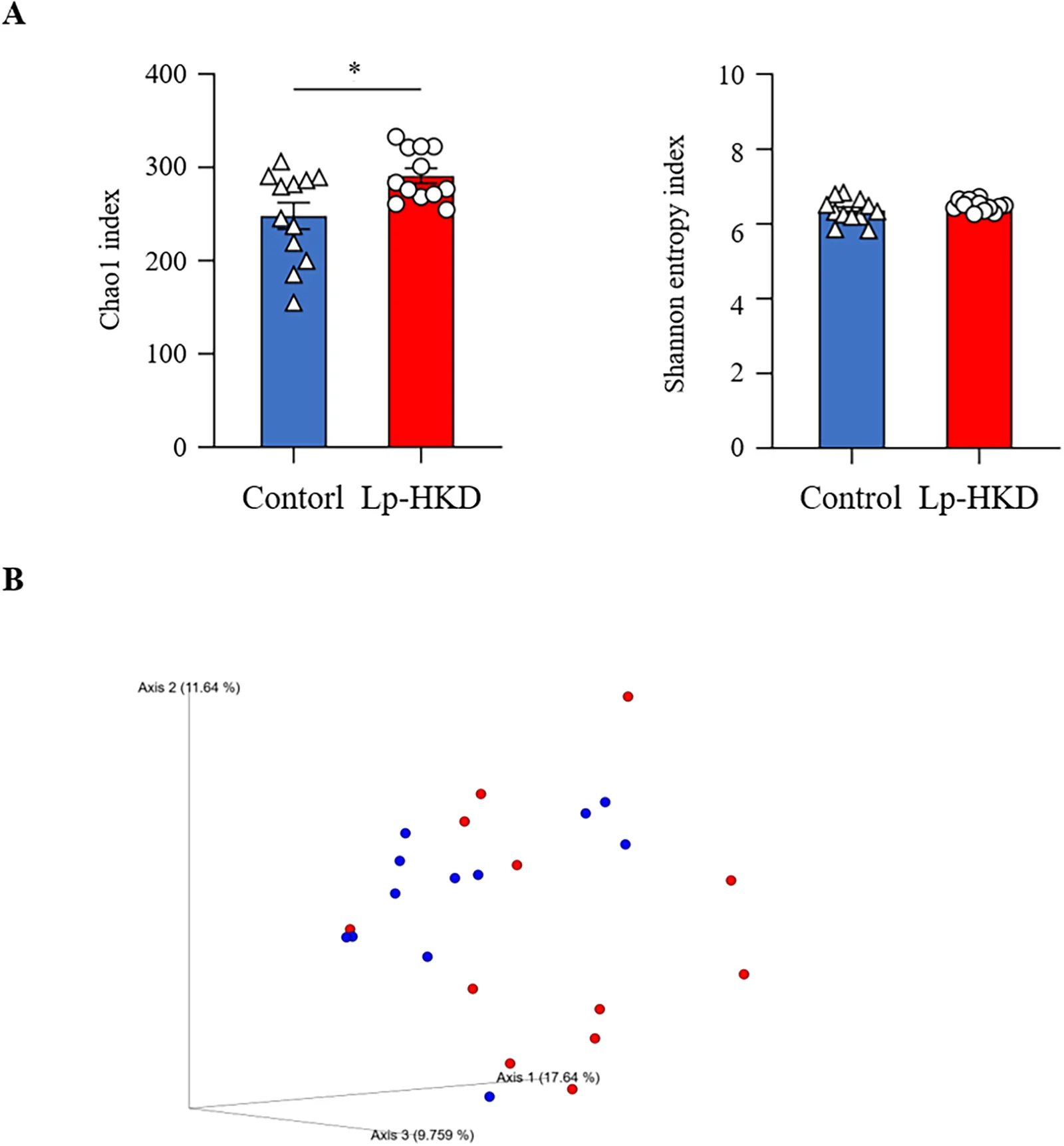
Lp-HKD-induced alterations in the gut microenvironment. **(A)** alpha diversity, assessed using the Chao1 and Shannon entropy indices (*n* = 12/group). **(B)** PCA of microbial community structures. Statistical significance was determined by the Welch’s *t*-test [**(A)** Chao1 index], PERMANOVA **(B)**, and Student’s *t*-test [**(A)** Shannon entropy index]. *p < 0.05.

### 3.6 Importance of alveolar macrophages in SeV infection and their modulation by Lp-HKD administration

Based on the association between alveolar macrophage numbers and reduced viral burden, we next investigated whether alveolar macrophages were required for Lp-HKD–mediated protection. Specifically, clodronate or control liposomes were administered intranasally 4 days before SeV infection (0.6 × 10^3^ TCID_50_). Subsequently, survival and body weight were monitored for 14 days post-infection (**Figure 8A**). Alveolar macrophages were depleted in the lungs 4 days after intranasal administration of clodronate liposomes (**Supplementary Figure S4**). As shown in **Figure 8B**, the survival rate of the control group dropped sharply to 60% on Day 9 and further declined to 50% by Day 14. Furthermore, depletion of alveolar macrophages in the control group further reduced survival beginning on Day 7, reaching 9% by Day 14. In contrast, the Lp-HKD group moderately decreased to 89% on Day 9 but maintained this survival rate through Day 14. However, when alveolar macrophages were depleted, survival among mice receiving Lp-HKD also markedly decreased from Day 7, declining to 27% by Day 14. **Figure 8C** shows the weights of mice in the Lp-HKD group began to stabilize around Day 5 and recovered from Day 6 onward. In contrast, the other groups continued to lose weight until Day 7. Although a subsequent recovery trend was observed, the extent of recovery was less pronounced than in the Lp-HKD group. Thus, alveolar macrophages are critical for protection against SeV infection, and this finding further suggests that Lp-HKD administration may enhance this protective effect.

**Figure 8:**
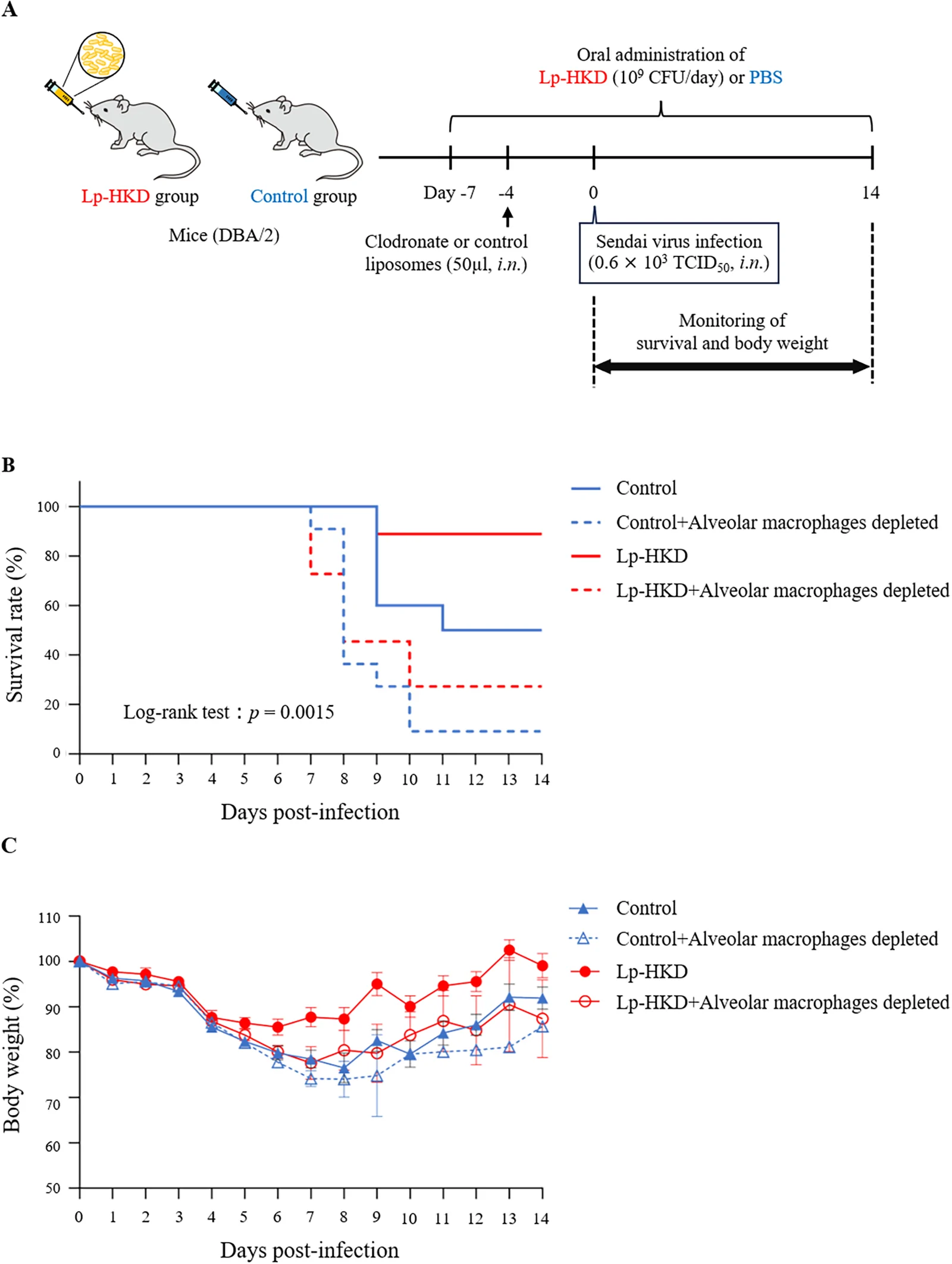
The role of alveolar macrophages in Lp-HKD-mediated protection during SeV infection. **(A)** Outline of the experimental scheme. DBA/2 mice (Lp-HKD group, *n* = 20; control group, *n* = 21) received daily intragastric administration of 100 µl of Lp-HKD (1 × 10^9^ CFU/mouse) or PBS (control) beginning 7 days before SeV infection (0.6 × 10^3^ TCID_50_) and continuing until Day 14 post-infection. 4 days before SeV infection, mice were intranasally administered 50 µl of clodronate liposomes (Lp-HKD group, *n* = 11; control group, *n* = 11) or control liposomes (Lp-HKD group, *n* = 9; control group, *n* = 10). **(B)** Survival rates after SeV infection. **(C)** Body weight changes expressed as percentages relative to body weight on Day 0. Statistical significance was determined by the Log-rank test **(A)** and Student’s *t*-test **(B)**. *p < 0.05.

## 4 Discussion

This study revealed that intragastric administration of Lp-HKD confers significant protection against SeV respiratory infection in mice by orchestrating a coordinated modulation of systemic and pulmonary immunity. Notably, Lp-HKD enhanced splenic immune activation prior to infection, as evidenced by increased leukocyte numbers and elevated expression of *IFN-γ* and *TNF*-α. These results indicate that Lp-HKD primes the host systemic immune system even before viral challenge, which may have facilitated the rapid antiviral responses observed after infection. A recent study on the probiotic strain *Lactobacillus reuteri* L_2_6 strain, found that it confers protection against viral infections in mice by modulating systemic immune responses (21). In the present study, the systemic immune priming observed is consistent with previous findings showing that Lp-HKD activates bovine monocytes and T cells and induces antiviral cytokines *in vitro* (11). Furthermore, Lp-HKD stimulates human dendritic cells to produce IL-6, IL-10, IL-12, and TNF-α (8), suggesting that this probiotic strain may exhibit immunostimulatory effects in multiple experimental systems.

A central discovery of the present study is the preservation of alveolar macrophages in Lp-HKD–treated mice during SeV infection, whereas they were markedly reduced in control mice. Alveolar macrophage preservation was associated with significantly lower viral RNA levels in the lungs, and correlation analysis revealed a negative association between alveolar macrophage abundance and viral burden. SeV impairs the intrinsic antiviral functions of macrophages, which promotes SeV infection severity (22, 23). When macrophage activity is inhibited, viral replication increases, worsening the pathological outcome. Furthermore, SeV belongs to the *Paramyxoviridae* family, a group of viruses that induce apoptosis in infected cells (24). SeV can also trigger apoptosis via IFN regulatory factor 3-dependent activation of caspase-3 and caspase-8 (25, 26), suggesting that SeV may induce similar apoptotic processes in alveolar macrophages. These findings suggest that Lp-HKD administration preserves alveolar macrophage abundance during SeV infection, thereby upholding antiviral defense in the lung. The sustained antiviral activity of alveolar macrophages likely inhibits excessive viral replication and mitigates tissue injury, contributing to the overall protective effect of Lp-HKD. Further studies will be required to clarify how alveolar macrophages in Lp-HKD–treated mice become functionally distinct from those in control mice. However, an alternative possibility is that reduced viral burden or lung injury may simply result in better preservation of alveolar macrophages. Taken together, these two possibilities highlight the need for further mechanistic studies to determine how Lp-HKD influences the maintenance and function of alveolar macrophages during SeV infection.

Depletion experiments further validated the functional relevance of alveolar macrophages. Removal of alveolar macrophages dramatically worsened survival outcomes in both control and Lp-HKD–treated mice, confirming their indispensable role in host defense against SeV infection. Notably, the protective effect of Lp-HKD was largely abolished in mice with depleted alveolar macrophages, indicating that the presence of these cells contributes to the protective response during SeV infection [similar to findings reported for heat-killed *Lactobacillus casei*, which also showed alveolar macrophage-dependent protection against influenza A virus (27)]. However, because functional characterization of alveolar macrophages was not conducted, their role in SeV infection cannot yet be defined, indicating that further mechanistic studies will be required. In addition, the slight improvement in survival among Lp-HKD mice with depleted alveolar macrophages compared with controls suggests that other immune pathways may also contribute to Lp-HKD–mediated protection.

In addition, beneficial effects of lactic acid bacteria on SeV infection have also been reported for strains other than Lp-HKD. For example, oral administration of *Lactococcus lactis* JCM5805 strain improves survival and reduces weight loss in SeV-infected mice by activating plasmacytoid dendritic cells and inducing type I and III IFNs, which subsequently enhance antiviral gene expression in the lung (28). These findings suggest that lactic acid bacteria can exert protective effects through immune pathways beyond those involving alveolar macrophages. Taken together with our results, these reports suggest that probiotic-mediated antiviral activity may operate through diverse immune pathways depending on the strain, highlighting the importance of clarifying strain-specific and tissue-specific mechanisms of action.

Another important aspect of the protective effect of Lp-HKD is its attenuation of pulmonary inflammation. While cytokines and chemokines are essential in pathogen elimination and immune cell activation, excessive production of inflammatory types significantly drives lung pathology during respiratory viral infections (29–33). In the present study, BALF cytokine or chemokine concentrations did not differ significantly between Lp-HKD–treated and control mice at Day 3 post-infection, with viral levels in the lungs showing a similar pattern and both groups exhibiting comparable viral loads at this early time point (data not shown). However, at Day 7 post-infection, clear differences emerged. Lp-HKD administration markedly reduced BALF levels of multiple cytokines (IFN-γ, TNF-α, IL-12p40, and IL-17A) and chemokines (KC, MCP-1, MIP-1α, and RANTES), while significantly lowering viral levels in the lungs during SeV infection. SeV viral loads tend to peak at Days 4–5 post-infection (34). Therefore, the observed reductions in viral burden and inflammatory mediators at Day 7 indicate that Lp-HKD enhances host-mediated viral clearance rather than suppressing early viral replication, potentially by modulating alveolar macrophage function. Because viral RNA levels also decreased at this time point, the lower cytokine and chemokine levels may reflect reduced viral burden and/or enhanced resolution of inflammation rather than direct suppression of inflammatory responses. This coordinated reduction in viral burden and inflammatory mediators may contribute to the improved clinical outcomes, including reduced weight loss and enhanced survival. Beyond these findings, the optimal dosing strategy and treatment duration for Lp-HKD remain important considerations for maximizing its protective efficacy. This study did not assess different doses or treatment durations of Lp-HKD, which may influence the magnitude of its antiviral and anti-inflammatory effects. Future work should examine dose–response relationships and compare short-term versus extended administration to identify the optimal regimen for achieving consistent protection.

Given the emerging interest in the gut–lung axis in respiratory immunity, we also examined the impact of Lp-HKD on the gut microenvironment. Although Lp-HKD administration altered the abundance of certain bacterial taxa and increased microbial richness, the overall microbial community structure remained largely unchanged. These modest alterations indicate that any microbiota shifts associated with Lp-HKD are limited and should be interpreted as exploratory findings rather than evidence of a causal gut–lung mechanism. The antiviral effects of Lp-HKD may instead arise from immune-modulatory pathways—such as pattern-recognition receptor signaling, or mucosal immune enhancement—rather than broad microbiota remodeling. Although we quantified multiple organic acids in cecal feces, the observed decreases in *n*-butyrate, acetate, and propionate levels in Lp-HKD–treated mice remain unresolved. These declines may reflect either insufficient organic acids or increased consumption within the system, and at present there remains room for debate regarding which mechanism predominates. Accordingly, these changes cannot yet be interpreted mechanistically, and their relevance to host responses remains to be determined.

Growing evidence indicates that gut-derived microbial signals influence pulmonary immune responses, partly by modulating the functional state of alveolar macrophages, which are central to maintaining lung health (35). For example, segmented filamentous bacteria remotely reprogram alveolar macrophages to enhance antiviral activity (36), while certain probiotics mitigate respiratory syncytial virus infection by modulating the microbe–alveolar macrophage axis (37). Although several mechanisms have been proposed to elucidate the influence of gut-derived microbial components on alveolar macrophage function, a substantial portion of this gut–lung communication remains poorly understood. In the present study, we could not determine the precise mechanisms by which Lp-HKD modulates alveolar macrophages. Thus, further analyses are required to clarify the influence of Lp-HKD on alveolar macrophage.

Collectively, our findings reveal that Lp-HKD confers enhanced resistance to respiratory viral infection through a multifaceted mechanism involving systemic immune priming, preservation of alveolar macrophages, suppression of excessive inflammation, and modulation of the gut microenvironment. Given calves’ high susceptibility to respiratory diseases and the previously demonstrated efficacy of Lp-HKD against bovine rotavirus, Lp-HKD could represent a promising approach for reducing viral infection risk in calves. Future studies should elucidate the molecular pathways underlying Lp-HKD–induced preservation of alveolar macrophages and compare its effects across other target animal species.

Finally, the translation of murine results to calves and humans presents inherent limitations. Species-specific differences in lung structure, immune responses, and gut microbial ecology may alter the efficacy and mechanisms of Lp-HKD. Thus, further studies in calves and well-controlled human trials are required to determine whether the protective effects observed in mice can be reproduced in target populations.

## Data Availability

The datasets presented in this study can be found in online repositories. The names of the repository/repositories and accession number(s) can be found in the article/**Supplementary Material**.
